# Relations of the Dental Pulp to Other Tooth Tissues

**Published:** 1878-09

**Authors:** L. C. Ingersoll

**Affiliations:** Keokuk, Iowa.


[TO BE CONTINUED.]

---

### ARTICLE II.

### *Relations of the Dental Pulp to the other Tooth Tissues.*

BY L. C. INGERSOLL, KEOKUK, IOWA.

Any attempt to pass judgment on the relative value of things essential to life or existence is as futile as the decision of the question in the school-boy lyceum, "Which is most essential to the well-being of man—air or water?" To decide the question on its merits, in favor of either side, is to nullify unanswerable arguments on the other side.

If we assume that the relation of the tissues of the teeth is as absolute as the component elements of air or water; if the tooth pulp is, at all ages of the individual and in all its vital conditions, a *sine qua non* of tooth structure, as hydrogen is of water, there is no debatable ground on which to plant a doubt. But, on the other hand, if it can be shown that it is not absolute and unchanging in its relations, but variable with age and its own vital conditions, we may pertinently ask, What are its changed conditions and its changed relations to the other tooth tissues, and how should the facts evolved modify our practice?

Dentists everywhere have encountered the popular opinion that when the nerve or pulp of a tooth dies the remaining tooth substance is soon lost. How generally this popular opinion has domiciliated itself in the minds of the dental profession is told in the universal interest which the subject of pulp preservation has elicited during the last twenty-five years. It was also made quite evident at the last meeting of the American Dental Association, when the writer of this paper broached the subject now offered for your consideration. We often find ourselves holding opinions of which we can give little or no account as to how we became possessed of them, except that such are the common belief. A hundred men will walk in the same devious path through the snow, without being able to give any reason for their course farther than that found in the fact that they are following the tracks of the first man that passed along that way after snow fall. Opinions, notions and tissues of thought are entailed upon us like many of our physical idiosyncrasies. We believe and adopt them because our seniors believed and adopted them. In this era of progress and evolution, a decade of years should not pass without a retrospect of our thoughts and opinions, to determine whether they are founded in fact or fancy. The learned among the Greeks handed down to their posterity fables as well as facts. In our day we have to deal with ancestral foibles rather than fables.

In undertaking to solve the problem of the functional value of the pulp at different periods in life, and in its varying vital and pathological conditions, I desire first to call your attention to a few accepted facts.

The function of the pulp in developing the tooth is unquestioned. But what does development mean? It means something more than presenting the dental organs to view in the mouth. In this sense an ear of corn with well-expanded kernels is fully developed, while yet the husks are green. But the corn is yet in the milk; and even after the glazing of the kernel with its yellow enamel, there is a hardening process going on within to bring it to maturity. Teeth when they cut the gum are yet in the "milk." They must gain in density by a ripening process, not so much chemical as functional. When the teeth first appear, the soft tissue bears a much larger proportion to the hard tissue than later in life. There *is* a period when the developing dental structure in all its form and substance is *wholly* soft or animal tissue. But from the moment an atom or a molecule of calcifying matter becomes fixed in the periphery of the pulpy mass, this new element is an entity claiming space. Space previously occupied by soft tissue is relinquished to the hard tissue. Though it is quite possible that by prolification of cells, the pulp for a while maintains its undiminished size, the limit of space renders it mathematically certain that at length the soft tissue must diminish in exact proportion as the hard tissue increases.

This supremacy of the hard tissue once gained, every month and every year lessens the size of the pulp by as much as the hard tissue has gained. *Mark well this tendency to obliteration of the pulp.* It is not a recession or retrogression under pathological influences, but under physiological law. When, therefore, you say that the dentine tubes are being filled up, or that the tube fibrils are being calcified, you are saying this—that the pulp is diminishing its area—that it is diminishing its territorial occupancy—that in the deserted territory the pulp has ceased its functions—that this is the process of pulp extinction. Every progressive step toward calcification of dentine is just so far a measure of the extinction of pulp substance and pulp function.

We all have observed that the pulp in the tooth of a youth is much larger than in the tooth of an adult. In harmony with this is the histological fact that the dentine tubes are much larger in early life than in adult life; also that other fact which has come to the observation of histologists, that odontoblast cells decrease in size and in number with the age of the tooth.

What is this steady decrease with advancing life, of the size of the dentine tubes, but a steady diminishing of the pulp and pulp influence? What is this increase of the mineral portion but a decrease of the animal and vital portion? What is this process of calcification of dentine fibrils, this encroachment of the walls of the pulp chamber upon the pulp itself, but progress towards pulp extinction? What is this process of mineralization of the teeth at the expense of the animal portion but an indication that the economy of nature is being so fully met *by mineralization* as to need less and less the functional power and nutrition of the pulp?

We have an example of more complete mineralization and annihilation of function in the enamel. It was once as wholly animal in its tissue, and as dependent upon vital functions, as the dentine. But by a process of more rapid development than dentine it passes almost wholly over from the organic kingdom to the mineral kingdom, and becomes in like degree independent of its early organic life. We thus see that the teeth occupy middle ground between the animal and the mineral kingdoms—neither belonging wholly to the one nor wholly to the other—being in their early organic nature wholly under the control of the animal functions, and in their later and more perfected state subject in greater part, to the laws of the mineral kingdom. This is an important view of the subject, as shedding some light on the relative importance of the pulp at different periods of development of the dental structure. With the change of substance comes the changed relation of the animal to the mineral portion of teeth.

The old physiologists numbered the teeth among the bones of the body. But when their peculiar structure came to be studied, it was found that neither physiologically nor pathologically could they be called bones. Comparisons cannot be justly instituted between them and bone, or any other internal structure. Teeth can more properly be classed with the dermal appendages. They are dermal *out*-growths and not *in* growths, and, like all out-growths, arrive at a measure of independence of that vitality so essential to all internal structures.

In the animal kingdom teeth are allied to horns, scales of fishes, the hard covering of some insects, and to all crustacea; and in their relation to vitality should be compared with these rather than mith bones invested with flesh.

For a single illustration take the horns of a deer. The deer's horn has both an inner and an outer pulp. What is known as the " velvet" is the outer pulp. This is highly vascular and wonderfully active in its formative function. Its vessels communicate with the inner pulp through the hardening tissue of the horn as the nerve fibrils of teeth ramify the dentine. As the soft horn becomes mineralized, the canals, through which the outer and inner pulps communicate, are gradually filled up, till at length the outer pulp is cut oft from its connection with the inner pulp, and dies. Then by the dictate of some animal instinct its bloody substance is rubbed off. Thus the outer portion of the horn is left without vital support and nutrition.

Tomes, in his Dental Anatomy, speaks of classes of teeth " in which the whole pulp is converted into solid material and no pulp cavity remains." [p. 79.] Then on the following page says: " The pulp of a sperm whale *becomes*

*obliterated* by a development of secondary dentine." In speaking of the tusk of the elephant, which is probably as nearly identical with human dentine as any animal formation, he says [p. 323 :] "The last remains of the pulp are converted into dentine in which a few vascular canals persist; these, of course, occupy the centre of the tusk, and are small in amount."

Of the teeth of rodentia, he says [p. 337 :] "The last remains of the pulps are converted into secondary or osteodentine, which thus forms the central axis of the incisors or molars, as the case may be. In this tissue vascular tracts sometimes exist, but altogether small in amount; the formation of true dentine going on till the pulp at that particular point is almost obliterated." On page 339 he says of the same: "Near to the surface actually in wear they [*i. e.*, the dentinal tubes] become cut off from the pulp cavity, by the conversion of what remains of the pulp into a laminated granular mass, so that the dentine exposed on the surface of a rodent's tooth must be devoid of sensitiveness, and the contents of the dentinal tubes must have presumably undergone some change; but what the nature of the change in the contents of dentinal tubes which have ceased to be in continuity with a vascular living may be, there are, so far as I know, no observations to indicate."

In his description of the teeth of the parrot-fish we read [p. 213 :] "When calcification has proceeded so far as to obliterate their central pulp, cavities, etc."

The above facts lead us to the conclusion that the developmental process in the formation of dentine is continuous and towards perfectibility, and that perfectibility of dentine means a calcification of dentinal fibrils so complete as to wholly exclude vitality from the area so calcified. Notice, too, this completeness of calcification is not the result of formative action excited at indefinite intervals by irritation from without, or by any external influence, but is *inherent in the nature of the function*, and is a continual progression according to physiological law.

In the mouths of some persons this perfectibility of structure is greatly retarded or wholly prevented by diseased conditions, general or local. Operations on such teeth are always attended with pain, even after middle life of the patient. But the experience, even of a limited practice, brings convincing evidence of teeth in the mouths of persons healthful organization that are entirely free from sensation throughout a large portion of their crowns, rendered so by a completed calcification. Such teeth are found in the mouths of many persons in middle life and on down to old age. We should be familiar with a larger number of cases of this kind, except that persons with such teeth seldom need the services of a dentist. By every test for vitality, such dentine, receiving no nutrient support from the pulp, is really dead. Yet every dentist reckons such teeth in the soundest possible condition, least disposed to decay. For this is the most complete tooth structure—complete in proportion to the obliteration of the pulp fibrils, thereby rendering such portion of the hard tissue independent of pulp influence.

